# Transplantation in the nonhuman primate MPTP model of Parkinson's disease: update and perspectives

**DOI:** 10.5194/pb-4-185-2017

**Published:** 2017-10-11

**Authors:** Florence Wianny, Julien Vezoli

**Affiliations:** 1 Univ Lyon, Université Claude Bernard Lyon 1, Inserm, Stem Cell and Brain Research Institute U1208, 69500 Bron, France; 2 Ernst Strüngmann Institute (ESI) for Neuroscience in Cooperation with Max Planck Society, 60528 Frankfurt, Germany

## Abstract

In order to calibrate stem cell exploitation for cellular therapy in neurodegenerative diseases, fundamental and preclinical research in NHP (nonhuman primate) models is crucial. Indeed, it is consensually recognized that it is not possible to directly extrapolate results obtained in rodent models to human patients. A large diversity of neurological pathologies should benefit from cellular therapy based on neural differentiation of stem cells. In the context of this special issue of Primate Biology on NHP stem cells, we describe past and recent advances on cell replacement in the NHP model of Parkinson's disease (PD). From the different grafting procedures to the various cell types transplanted, we review here diverse approaches for cell-replacement therapy and their related therapeutic potential on behavior and function in the NHP model of PD.

## 1 Introduction

The term Parkinson's disease (PD) makes reference to an ensemble of neurodegenerative conditions affecting several parts of the brain (Braak et al., 2006). PD is defined by the presence of α-synuclein positive inclusions into cell bodies and dendrites of monoaminergic cells, associated with the principal pathologic characteristic which is progressive death of pigmented cells of the substantia nigra pars compacta (SNpc), i.e., nigrostriatal dopaminergic (DA) neurons. These DA neurons disappear with an annual estimated rate of 1 % in parkinsonian patients compared to 0.5 % in healthy subjects (Scherman et al., 1989). Characteristic clinical signs appear late, i.e., when neuronal death exceeds the threshold of 70–80 % of nigrostriatal denervation and 50–60 % of neuronal death in SNpc (Agid, 1991). However, PD diagnosis is mainly clinical and based on a characteristic motor phenotype, i.e., bradykinesia, resting tremor, muscular rigidity, postural instability and freezing of gait. The presence of these motor troubles is used for the primary diagnosis of a parkinsonian syndrome; additional exclusion–inclusion criteria allow clinicians to differentiate between several forms of parkinsonism including PD – e.g., clinical motor symptoms generally have unilateral onset in PD (Chia and Liu, 1992). Several possible treatments are currently available for both early and late stages of the disease. However, PD remains incurable and those palliative therapies give rise to complications after several years of treatment. At present, symptomatic treatments of PD involve mainly L-DOPA (levodopa, L-3,4-dihydroxyphenylalanine) therapy for correcting motor symptoms and deep brain stimulation reserved for a subpopulation of patients. Nonetheless, these therapeutic approaches are not fully satisfactory because, even if movements are better controlled, they (i) do not cure the source of these motor and non-motor symptoms, (ii) do not prevent the disease progression and (iii) lead in the long term to behavioral troubles (e.g., impulse control disorders) at a significant rate (Destee, 2005).

A better comprehension of the physiopathology of PD and the establishment of new therapies requires an in-depth investigation of early stages of the disease, including pathophysiological characterization of (i) evolving non-motor symptoms, (ii) the restructuring of the central nervous system induced by DA lesion initiation and (iii) non-motor behavioral manifestations of the disease. Early onset of cognitive troubles linked to PD is now recognized (Yang et al., 2016), and they are in part due to fronto-striatal loop dysfunction (Brown and Marsden, 1990; Raskin et al., 1990; Owen et al., 1992) that can degenerate into psychological and behavioral troubles, e.g., dementia, with psychiatric symptoms being identified in 40–80 % of patients presenting cognitive deficits (Greene et al., 1993; Oikonomou and Paparrigopoulos, 2015). Early sleep and circadian disorders have been increasingly observed in the majority of patients (Adler and Thorpy, 2005), concerning many physiologic circadian rhythms (Bruguerolle and Simon, 2002). Thus, it appears essential to precisely study the evolution of neuronal reorganization at play during early stages of the disease for the identification of new therapeutic targets and also to characterize the multiparametric impact of therapeutic treatment on motor and non-motor aspects of PD.

The study of behavioral, physiological, anatomical and biochemical consequences of DA neuronal death in the basal ganglia was greatly facilitated by the availability of neurotoxins capable of inducing a highly selective death of DA neurons in animals, e.g., 1-methyl-4-phenyl-1,2,3,6-tetrahydropyridine (MPTP) or 6-hydroxydopamine (6-OHDA) for primate and rodent models (Burns et al., 1983; Langston and Ballard, 1984; Riachi et al., 1988; Lange, 1990; Forno et al., 1993; Nerobkova et al., 1996; Asakawa et al., 2016; Franke et al., 2016).

These animal models of PD have been and are still essentials for the development and amelioration of new therapeutic strategies. It is now well acknowledged that testing in nonhuman primate (NHP) models is a safe and requisite preclinical step before any translation to the clinic of brain transplantation. NHPs are more appropriate than non-primates for in vivo screening, because of their relative closeness to humans, notably regarding brain organization. In particular, primates share precise cortical development phases and associated compartmental growth of inter-areal connections (Kennedy and Dehay, 2012); late frontal lobe development, both phylogenetically and ontogenetically; compartmentalization of meso-frontal projections according to a mediolateral gradient (Williams and Goldman-Rakic, 1998; Raghanti et al., 2008); and functional organization of cortical and subcortical striatal afferents (Haber, 2003). All these features are critically involved in the evolution and manifestation of PD at different stages.

The low-dose MPTP monkey model (Bezard et al., 1997) is the model of choice for translational study because it presents a parkinsonian syndrome characterized by all critical aspects of PD, including a slow progressive evolution of symptoms, e.g., it replicates the typical motor symptoms used for primary clinical diagnosis of parkinsonism with response to classical DA therapy (Stephenson et al., 2005), characteristic pattern of nigrostriatal denervation observed in PD patients (Gibb and Lees, 1991; Perez-Otano et al., 1994), early and long-lasting non-motor symptoms (Poewe, 2008; Vezoli et al., 2011; Fifel et al., 2014; Swallow et al., 2016), and increased α-synuclein expression in the pigmented cells of the SN (substantia nigra; Purisai et al., 2005). However, even if those inclusion bodies appears in the same sites as for Lewy bodies in PD, e.g., SN, they do not express typical Lewy body features as found in human PD patients (Forno et al., 1993).

MPTP rodent models have been useful to unravel mechanisms underlying DA neuron loss; however, their DA system and sensitivity to MPTP vary highly across species (Jossan et al., 1989; Sundstrom and Samuelsson, 1997) and differ from primates (Johannessen et al., 1985). One explanation is that rodents express extremely small amounts of neuromelanin (DeMattei et al., 1986), which is abundant in human brainstems and is proposed to have a role in long-lasting toxicity of MPTP in primates (Jackson-Lewis and Przedborski, 2008).

The NHP MPTP model hence reproduces a large repertoire of motor and non-motor impairments found in PD patients and is thus perfectly suited for a multiparametric evaluation of the therapeutic efficacy of cell transplant as well as for developing and refining techniques for improving integration of grafted cells and minimizing potential side effects of the graft. Here we focus on preclinical investigations done in the gold standard NHP MPTP model (Emborg, 2007; Potts et al., 2014). We provide a non-exhaustive review of different procedures using transplantation of various cell sources as a therapeutic approach in the NHP MPTP model of PD and provide an update on linked therapeutic behavioral and functional outcomes.

## 2 The grafted cells: types and characteristics

The first attempts to treat PD with cell replacement date back to the early 1970s, with the idea of reestablishing striatal DA transmission and restoring a regulated release of DA in the striatum. Two different cell sources for auto- and allografts (adrenal medullary tissue and ventral mesencephalic region of fetuses respectively) paved the way for brain repair through cell transplantation. Adrenal medulla grafts which contained chromaffin cells that synthesized DA were grafted in 6-OHDA rats as an alternative source of catecholamine-producing cells (Freed et al., 1981; Stromberg et al., 1985) with the benefit of avoiding ethical and immunological issues linked to the graft of fetal tissue. Even if testing in the 6-OHDA NHP model returned minimal survival of transplanted adrenal medullary tissue (Morihisa et al., 1984), it was rapidly followed by first clinical trials in the few patients reporting clinical improvement (Lindvall et al., 1987; Madrazo et al., 1987). However, grafts in larger cohorts of PD patients returned rather unsatisfactory results, calling for more careful investigations at the preclinical level (Sladek Jr. and Shoulson, 1988).

### 2.1 Fetal ventral mesencephalon

The fetal ventral mesencephalon (fVM), which generates DA neurons during development, constituted another promising candidate for cell grafting. In the late 1970s, transplantation of rodent fVM tissue into the lateral ventricle adjacent to the putamen of 6-OHDA-lesioned rats as an alternative to L-DOPA treatment was first performed (Perlow et al., 1979) and showed that fVM tissue could survive, innervate the host striatum, release dopamine and reverse many of the behavioral deficits in this PD model. The functional and behavioral outcome of brain transplantation was then investigated further in 6-OHDA rats for several years until the first trial of transplanting human fetal DA neurons was performed in the same rodent model (Brundin et al., 1986). At the time, testing of fVM striatal grafts on the recently developed NHP MPTP model (Burns et al., 1983) returned satisfactory results, with behavioral recovery in monkeys exhibiting mild to severe parkinsonism (Redmond Jr. et al., 1986; Sladek Jr. et al., 1988; Annett et al., 1990, 1994, 1995; Taylor et al., 1991; Starr et al., 1999; Collier et al., 2002), confirming trials in the rodent model of PD. First clinical trials (Lindvall et al., 1988) were again initially promising (Lindvall et al., 1990) but were later held back due to efficiency concerns following studies with a larger number of patients and randomized, double-blind, placebo-controlled protocols (Freed et al., 2001; Olanow et al., 2003) that showed no significant difference between grafted patients and placebo, with several patients developing graft-induced dyskinesia. Nevertheless, retrospective analyses and reports on those clinical trials of fVM striatal grafts demonstrated their efficiency either clinically (Kefalopoulou et al., 2014), histologically (Li et al., 2016), behaviorally (Gordon et al., 2004) or functionally (Politis and Piccini, 2010). Based on all these evidences, a new clinical trial of fVM transplantation has been started on a cohort of patients with improved grafting procedures and selection of patients (Moore et al., 2014) with the aim of preparing for the move to stem-cell-based transplantation in humans while waiting for preclinical investigation reports in animal models.

The development of the MPTP NHP model of PD contributed enormously to the standardization of the methodology, due to the possibility of studying large cohorts of monkeys – in particular, in determining the optimal stage of the donor fetuses (Sladek Jr. et al., 1993b; Elsworth et al., 1996), the conditioning of the grafted sample, the site of transplantation (Collier et al., 2002) and the mechanism of graft-induced recovery (Bankiewicz et al., 1990). More recently, transplantation methods and sites of placement of the transplanted cells were revisited and refined in the NHP model (Redmond Jr. et al., 2008; Kordower et al., 2017).

All the transplantation studies performed with fVM in NHPs were based on the assumption that fVM was enriched in immature DA neurons, although most of the studies were performed without any detailed analyses of the cellular content of the grafted tissue. However, fVM contains a heterogeneous population of cells whose composition fluctuates during development and comprises a relatively low proportion of DA progenitors divided in two subtypes: (1) progenitors that will give rise to A9 neurons of the SNpc that express the G-protein-gated inwardly rectifying K+ channel, GIRK2; and (2) progenitors that will give rise to A10 neurons of the ventral tegmental area which express the calcium binding protein, CALBINDIN (Thompson et al., 2005). A9 neurons are the essential functional components for recovery of motor function in rodent models of PD (Kuan et al., 2007; Grealish et al., 2010). Apart from DA progenitors, fVM also contains a high diversity of radial glial cells; other types of progenitors, including serotonin, GABAergic and oligodendrocyte precursors; and non-neural cell types, such as endothelial cells, pericytes and microglial cells (La Manno et al., 2016). This variability in tissue composition as well as other issues, including limited availability of fetal brains and ethical concerns associated with the use of aborted fetal tissues, make it very difficult to generalize this cell therapy approach.

Progress in the field of stem cells brings hope that this type of cell therapy could be generalized to treat PD patients. A number of pluripotent stem cells (PSCs) have been tested in NHPs, isolated either from early stage embryos (embryonic stem cells, ESCs) or from reprogrammed somatic cells (induced pluripotent stem cells, iPSCs). PSCs have the capacity to become any cell types in the body, including dopaminergic progenitors and neurons. They thus constitute an infinite source of cells for transplantation into PD patients.

We will now focus on the transplantable DA cell types generated from primate PSCs, which represent the closest to clinical application. Human ESCs (Kriks et al., 2011; Daadi et al., 2012; Doi et al., 2012; Grealish et al., 2014; Gonzalez et al., 2015, 2016; Chen et al., 2016) and monkey ESCs (Kawasaki et al., 2002; Sanchez-Pernaute et al., 2005; Takagi et al., 2005; Xi et al., 2012) were first used, recently followed by human iPSCs (Kikuchi et al., 2011; Kriks et al., 2011; Sundberg et al., 2013; Doi et al., 2014) and monkey iPSCs (Morizane et al., 2013; Sundberg et al., 2013; Wang et al., 2015).

### 2.2 DA neurons isolated from primate PSCs or by direct reprogramming of somatic cells

Various protocols available for the generation of DA neurons from human and NHP PSCs were adapted from those developed with mouse ESCs (Kawasaki et al., 2000; Lee et al., 2000; Watanabe et al., 2005). Early protocols aimed at first inducing neural differentiation of PSCs generally by culturing the PSCs with stromal cells (PA6 cells or MS5 mouse lines) or in the presence of medium conditioned by these cells (Takagi et al., 2005). Other protocols for neural differentiation involved suspension cultures to generate embryoid bodies and culture in serum-free medium (Roy et al., 2006; Iacovitti et al., 2007). These protocols enable a significant enrichment of the population into neural progenitors that expressed NESTIN, SOX1, PSA-N-CAM (polysialylated neural cell adhesion molecule), PAX6 and SOX2 (Kawasaki et al., 2002; Ben-Hur et al., 2004; Perrier et al., 2004; Park et al., 2005; Sanchez-Pernaute et al., 2005; Takagi et al., 2005; Vazin et al., 2008; Doi et al., 2012).

Midbrain DA specification of these neural precursors can then be induced by addition of FGF8, a mid- and hindbrain organizing morphogen, and SHH, a ventralizing morphogen (Perrier et al., 2004; Zeng et al., 2004; Park et al., 2005; Yan et al., 2005; Yang et al., 2008; Cooper et al., 2010; Doi et al., 2012), and/or by treatment with FGF2 and FGF20 – a secreted protein that enhances the survival of primary DA neurons (Ohmachi et al., 2000; Takagi et al., 2005; Morizane et al., 2013). Characterization of the cells showed that DA neurons express midbrain DA neuron markers such as NURR1 and LMX1A, LMX1B, FOXA2, OTX2, CORIN, PITX3, factors that control specification and differentiation of midbrain DA neurons during mouse development (reviewed in Arenas et al., 2015), and GIRK2, which is the A9-specific marker (Thompson et al., 2005). They also express tyrosine hydroxylase (TH), and the dopamine transporter (DAT), and they produce dopamine, confirming that they are functional DA neurons (Kriks et al., 2011; Kirkeby et al., 2012; Arenas et al., 2015).

Although these methods enabled efficient DA differentiation, the cultures usually comprise a high percentage of glial cells and multiple neuron subtypes, such as GABAergic, cholinergic and serotonergic neurons (Emborg et al., 2013b; Morizane et al., 2013). Complete and robust midbrain specification was recently obtained via a floor plate intermediate stage from human (Kriks et al., 2011; Xi et al., 2012; Sundberg et al., 2013) and NHP PSCs (Xi et al., 2012; Hallett et al., 2015; Wang et al., 2015), using a modified dual-SMAD inhibition protocol (BMP/TGFβ inhibition; Chambers et al., 2009) and activation of Wnt signalling, an essential pathway in DA neuron development in the mouse (Castelo-Branco et al., 2003, 2004; Joksimovic et al., 2009) and in humans (La Manno et al., 2016). Combining suspension culture with dual-SMAD inhibition, Wnt and SHH activation also led to robust VM differentiation, with correct midbrain GIRK2+ A9 and CALBINDIN+ A10 phenotypes, similar to fVM content (Kirkeby et al., 2012; Morizane et al., 2013; Doi et al., 2014; Grealish et al., 2014; Chen et al., 2016).

Most of the protocols to generate DA neurons from primate PSCs assume that primate VM development follows the same sequences of events and thus expresses the same set of specific markers as in rodents (recently reviewed in Arenas et al., 2015). However, major differences in human and rodent ventral midbrain development have recently been identified (La Manno et al., 2016). In light of these results, studies have been engaged to develop and refine the existing protocols and tools to generate DA neurons from primate PSCs in vitro that better mimic their in vivo counterparts. In this line, identification of a set of new markers of human mesencephalic DA progenitors (EN1, SPRY1, PAX8, CNPY1 and ETV5) enabled Kirkeby and collaborators to develop a differentiation protocol that leads to increased yield of DA progenitors from human ESCs (Kirkeby et al., 2017). These markers enable proper prediction of the resulting DA neuron content in the graft and will enable further standardization of DA differentiation protocols from PSCs.

These last generation of PSC-derived DA progenitors are produced under careful Good Manufacturing Practice (GMP) laboratory conditions (Kirkeby et al., 2017) and bare authentic fVM features: they are able to reinnervate the lesioned striatum and function with equal potency to fVM tissue upon striatal transplantation in the PD rodent brain (Kriks et al., 2011; Doi et al., 2014; Grealish et al., 2014; Chen et al., 2016). Based on recent preclinical in vivo assessments mostly performed in the rodent model and more recently in the NHP model, several clinical trials have been recently launched worldwide using human ESC DA cells, such as the European trial “STEM-PD” (Kirkeby et al., 2017).

DA neurons can also be generated by direct reprogramming of somatic cells such as mouse and human fibroblasts, using overexpression of different transcription factors and midbrain-specific factors such as LMX1A, ASCL1, NURR1, BRN2, FOXA2, NGN2, SOX2 and MYT1L (Caiazzo et al., 2011; Pfisterer et al., 2011a, b; Liu et al., 2012). These induced DA (iDA) cells led to reduction of motor symptoms when transplanted in the striatum of PD rodent models (Kim et al., 2011; Liu et al., 2012; Dell'Anno et al., 2014; Rivetti di Val Cervo et al., 2017) and represent an interesting cell source that still needs to be pre-validated in the NHP model.

The intended application of reprogrammed cells (either iPSCs or iDA cells) and their derivatives is autologous transplantation. However, their isolation and deep characterization usually needs several months, which constitutes an important drawback when considering that transplantations have to be done when the system is still reparable. Furthermore, because they are derived from PD patients, they may present genetic and epigenetic alterations and may be more susceptible to the pathological processes induced in PD.

### 2.3 Neural stem cells

Multipotent neural stem cells (NSCs) present several advantages over DA progenitors or neurons. They constitute homogeneous cell populations that can be expanded on a very large scale and extensively characterized, and they can be kept as frozen stocks that are ready to use for transplantation. This ensures high levels of batch-to-batch consistency and enables optimal traceability of the grafted cells. Another advantage of NSCs is that they are capable of giving rise to neuronal and non-neuronal progenies, such as astrocytes and oligodendrocytes. They are thus potentially capable of responding and adapting to the host environment, making them one of the best candidates to restore a functional equilibrium in the nigrostriatal system (Redmond Jr. et al., 2007).

Human NSCs have been isolated from fetal brains (Flax et al., 1998; Sun et al., 2008; Wakeman et al., 2009) or from PSCs (Gonzalez et al., 2016) through adaptation of the protocol used to derive mouse NSCs (Conti et al., 2005; Pollard et al., 2008). They express the stem cell markers NESTIN, SOX2, VIMENTIN and MUSASHI; are karyotypically normal; and can be amplified for long periods of time in culture without losing their properties (Redmond Jr. et al., 2007; Bjugstad et al., 2008; Wakeman et al., 2014; Gonzalez et al., 2015, 2016). Following testing for various contaminants (virus, mycoplasma, bacteria, etc.), they constitute working cell banks prepared under GMP-grade conditions (Gonzalez et al., 2015, 2016). NSC lines can further be carefully preselected for transplantation on the basis of their capacity to differentiate into DA progenitors in response to DA induction in vitro and to give rise to various neuronal and glial cell types upon transplantation into different regions of the mouse brain in vivo (Redmond Jr. et al., 2007).

Human NSCs have shown very promising results upon injection in SN and striatum of MPTP monkeys (Redmond Jr. et al., 2007; Bjugstad et al., 2008; Gonzalez et al., 2015, 2016). Although transplantations of human cells into the brain of NHPs bring crucial information, NSC safety and efficiency within an NHP species still need to be confirmed. This would imply the isolation and deep characterization of NHP NSC lines that are still sparse (Wianny et al., 2011).

### 2.4 Other cell types

Autologous neural cell ecosystems (or ANCE) represent an interesting alternative for autologous transplantation. These cells are derived from the adult monkey cortex; they do not require genetic modification and can be expanded in only a few weeks in vitro (Brunet et al., 2009; Bloch et al., 2014). They do not express specific DA markers, but progenitor markers such as GFAP (glial fibrillary acidic protein), NEUROFILAMENT, VIMENTIN and NESTIN (Brunet et al., 2009). It is still unclear whether these cells are able to differentiate into DA neurons (Bloch et al., 2014). They showed very good survival and integration upon auto transplantation in striatum and SN of the PD NHP brain, which is associated with behavioral recovery (Bloch et al., 2014). However, this system needs careful characterization in the NHP model to evaluate its usefulness in future clinical application.

Human retinal pigment epithelial (hRPE) cells may constitute another suitable cell type for transplantation in PD. They are isolated from the inner layer of the retina of postmortem fetal eyes and produce L-DOPA as a precursor of their characteristic eumelanin pigment through the activity of TH. They can be easily expanded in culture and stored as frozen stocks, allowing extensive characterization and testing before transplantation. hRPE cells attached to gelatin microcarriers (GM) show enhanced survival and improve motor deficits after striatal grafts in rodents (Subramanian et al., 2002; Cepeda et al., 2007) and in NHP models of PD (Watts et al., 2003; Doudet et al., 2004). RPE-GM are currently used in human PD patients under the name of Spheramine (Titan Pharmaceuticals, Inc.). The mechanism of action of RPE cells may not be the production of dopamine, but constant in situ release of low physiological level of levodopa, which may stimulate DA synthesis from surviving DA neurons.

In 2011, the results of the first randomized, double-blind, placebo-controlled trial of RPE cell transplantation in PD patients showed that hRPE cells provided no antiparkinsonian benefits over sham surgery (Gross et al., 2011). Optimal therapeutic benefit could be reached after administration of low doses of levodopa. These results have been recently confirmed in NHPs where unilateral striatal transplantation of hRPE-GM was not sufficient to completely reverse the motor symptoms of induced parkinsonism (Peng et al., 2016). Studies performed in the NHP model will be crucial to refining the characteristics of these RPE cells used for transplantation, in particular the optimal age of the donors. Fetal hRPE cells indeed showed higher survival than those obtained from neonatal donors, at least in the rat PD model (Peng et al., 2016; Russ et al., 2017). The NHP model may also help in evaluating the therapeutic effect of other sources of hRPE cells and other formulations of microcarriers to increase cell survival (Falk et al., 2012).

### 2.5 Cell labeling

As far as xenografts are concerned, human-grafted cells are easily distinguished from the host primate brain using human-specific markers, such as the human cytoplasmic markers hCy (Daadi et al., 2012) and STEM121 (Kriks et al., 2011; Gonzalez et al., 2015; Chen et al., 2016). In contrast, localizing the NHP cells upon transplantation in NHP models necessitates labeling cells before grafting.

fVM tissue cannot be labeled prior to transplantation; its progenies are thus commonly detected by simple TH immunofluorescent staining. This strategy enables locating the transplanted tissue, but the migration of the grafted cells and connection with host TH+ cells cannot be monitored (Taylor et al., 1991; Sladek Jr. et al., 1998). Furthermore, the high density of DA neurons in the core graft often precludes accurate counting (Brundin et al., 1986; Sladek Jr. et al., 1998). As previously mentioned, the developing human VM also contains others types of progenitors that may not give rise to TH+ neurons (La Manno et al., 2016). Thus, TH+ labeling may underestimate the variety of progenies arising from fVM grafts.

In contrast to fVM tissue, NSCs, DA progenitors and DA neurons can easily be labeled in vitro, prior to grafting. This was originally performed through incorporation of BrdU (bromodeoxyuridine; Takagi et al., 2005; Redmond Jr. et al., 2007, 2008). The disadvantage of this labeling is that not all cells become labeled with BrdU, leading to underestimation of cell survival (Takagi et al., 2005; Redmond Jr. et al., 2007). With the emergence of new technologies, cells are now frequently labeled with fluorescent proteins (GFP or RFP) after electroporation of a GFP-expressing plasmid and antibiotic selection (Morizane et al., 2013), or lentiviral infection (Gonzalez et al., 2016). Although the efficiency and stability of the labeling were not always documented, this type of labeling remains essential to assess the migration and facilitates visualization of neurite extension of the grafted cells (Gonzalez et al., 2016). The fluorescent dies PKH26 and PKH67 can also be cited (Bloch et al., 2014; Wolff et al., 2015), but their propensity to fade overtime makes them inappropriate for long-term labeling.

So far, GFP and RFP labeling seems most appropriate for evaluating integration and differentiation rates of grafted cells. However, some studies reported GFP-induced cytotoxicity (Liu et al., 1999; Detrait et al., 2002) and, more recently, dose-dependent toxicity in DA neurons in rats after cytoplasmic GFP transfection (Klein et al., 2006; Ansari et al., 2016). While this toxicity seems to be more associated with the level of expression of this reporter gene in transfected cells, this should be carefully taken into account while evaluating the efficacy of GFP-expressing transplant in NHPs.

It is noteworthy that senescent cells found in the aged and degenerative brain produce autofluorescence that interferes with detection of specific fluorescent signals, implying rigorous manual tracing analyses of transplanted cells in the case of fluorescent labeling (Spitzer et al., 2011; Salmonowicz and Passos, 2017).

Noninvasive methods developed for tracking cells in real time after grafting might also help to improve the design of future clinical cell transplantation. A nice example is cell labeling with superparamagnetic iron oxide (SPIO) nanocomposites, which allow cell tracing in living animals using MRI (Guzman et al., 2007).

## 3 Survival, differentiation and integration of the grafted cells

### 3.1 Number of grafted cells and survival

In studies performed with fVM, transplanted cells in the striatum consist of small pieces of fVM tissue (1 to 2 mm${}^{3}$; Elsworth et al., 1996; Collier et al., 1997; Leranth et al., 1998; Redmond Jr. et al., 2013) or cell suspensions that are usually not unicellular (Annett et al., 1994, 1995). The amount of grafted fVM tissue per monkey is the equivalent of 2–3 fetuses, which does not allow for precise determination of the number of transplanted cells (Table 1a). Consequently, the survival rate is also difficult to precisely evaluate. In contrast, single-cell suspensions of NSCs, DA progenitors or neurons enable precise counting of the transplanted cells, and 1 to 10 million of cells were usually injected per monkey brain (Table 1a).

Looking back on several decades of transplantation studies in MPTP NHPs, it appears that the survival rate of transplanted DA neurons is disappointing, with generally more than 90 % of the cells dying after transplantation (Table 1a; Sanchez-Pernaute et al., 2005; Redmond Jr. et al., 2007; Doi et al., 2012; Emborg et al., 2013a; Wang et al., 2015).

It is noteworthy that early DA neurons tend to survive better upon grafting in the brain of PD monkeys than do terminally differentiated DA neurons, as previously shown in rodents (Brundin et al., 1986). For example, VM tissue isolated from early stage fetuses showed a higher survival rate than that of older stage fetuses (Sladek Jr. et al., 1993b; Elsworth et al., 1996; Collier et al., 2002), as was previously shown in rodents (Fricker et al., 1997; Torres et al., 2007; Torres et al., 2008). Similarly, early DA progenitors derived from primate PSCs (D21–D28 neurospheres) produced larger grafts (Kikuchi et al., 2011) and showed a higher survival rate (Takagi et al., 2005; Wang et al., 2015) than did late DA neurons (D42 neurospheres; Sanchez-Pernaute et al., 2005; Takagi et al., 2005; Kikuchi et al., 2011; Doi et al., 2012; Emborg et al., 2013a). Nevertheless, the survival rate never exceeded 5 %.

In contrast to DA neurons, uncommitted neural cells show a much higher survival rate that is generally close to 10 % for NSCs (Redmond Jr. et al., 2007; Bjugstad et al., 2008; Gonzalez et al., 2015, 2016) and over 40 % in the case of autografts of immature neural cells derived from the adult cortex (ANCE; Bloch et al., 2014). The higher survival rate of immature cells, as compared to that of terminally differentiated neurons, could be explained by their multipotent features, which allow them to best adapt to environmental fluctuations. They also exhibit minimal neurite outgrowth and may thus be less sensitive than differentiated cells to mechanical stress during their isolation and transplantation procedure.

Interestingly, increasing the number of grafted cells did not improve cell survival proportionally in MPTP NHPs, regardless of their origin (fVM or PSC derivatives; Sladek Jr. et al., 1998; Bjugstad et al., 2008; Bloch et al., 2014; Hallett et al., 2015; Gonzalez et al., 2016). It has been hypothesized that a high cell number might induce host rejection in the case of allotransplantation and that it may also exhaust the supply of neurotrophic factors that are present in low amounts in the adult diseased brain.

**Table 1:** Grafted cells characteristics pre-graft.

**(a)**: Grafted cell								
characteristics								
pre-graft.								
Grafted	Origin	Developmental	Characteristics	Grafted number	Labeling	Recipient	Grafted	References
cell type		differentiation stage				species	sites	
Fetal fVM	fVM (NHP)	E74 (cell suspension)	not shown	1 VM/brain (2 µL/site)	NO	marmoset	CdN, Put, NAcc	Annett et al. (1994, 1995)
	fVM (NHP)	late fVM	not shown	1 VM/brain	NO	AFG	striata	Redmond Jr. et al. (1986)
	fVM (NHP)	E40–E50 & late	not shown	1 VM/brain (1 mm${}^{3}$ pieces)	NO	AFG	CdN	Sladek Jr. et al. (1993b);
		gestation		(6 sites/brain)				Elsworth et al. (1996a)
	fVM (NHP)	E44–47	not shown	1/6 VM/site (6 sites/brain)	NO	AFG	CdN or Put	Elsworth et al. (1996b);
								Collier et al. (1997)
	fVM (NHP) & co-grafts	E41–47	not shown	1/6 VM/site (1–2 mm${}^{3}$ pieces)	NO	AFG	CdN or Put	Sortwell et al. (1998)
	fVM/embryonic striatum							
	fVM (NHP)	E40–42	not shown	2 VM/brain (6 sites/brain)	NO	AFG	striatum	Sladek Jr. et al. (1998)
	fVM (NHP)	E43–47	not shown	1/2 VM/brain	NO	AFG	SN	Collier et al. (2002)
	fVM (NHP)	E44–45	not shown	1.5 VM/site	NO	AFG	Put	Kordower et al. (2017)
	fVM (NHP)	transfected with AAV5-	not shown	not shown	NO	AFG	CdN and Put	Redmond Jr. et al. (2013)
		hu-GDNF vector						
	fVM (NHP)	E40	not shown	multiple sites (1 mm${}^{3}$)	NO	AFG	CdN	Leranth et al. (1998)
	fVM (NHP)	E42 to E45 (cell	not shown	1.5 fVM/brain	NO	AFG	CdN or Put	Redmond Jr. et al. (2008)
		suspension/solid pieces)						
	fVM (NHP)	various stages	not shown	4 to 6 sites/brain (1 mm${}^{3}$)	NO	AFG	CdN	Taylor et al. (1991)
	fVM (NHP; early/late)	E38–44; E80–165	not shown	6 sites/brain (1 mm${}^{3}$)	NO	AFG	CdN or Put	Taylor et al. (1996)
	fVM (NHP)	E45–47; E52–54	not shown	1/2 VM/SN and 1 VM/striatum	NO	AFG	Put and SN	Sladek Jr. et al. (2008)
	fVM (porcine)	not shown	not shown	10 VM (1 mm${}^{3}$)	NO	cyno	Put	Aaron-Badin (2016)
	(CTLA4-Ig expression)							
NSCs	fetal brain (human)	NSCs at early passages (13GW)	Stable karyotype, proliferating SOX2+,	10${}^{6}$ cells/site	BrdU, LacZ	AFG	CdN, SN	Redmond Jr. et al. (2007);
			NESTIN+, VIMENTIN+, MUSASHI+					Bjugstad et al. (2008)
			Screened in vivo in mice					
	fetal brain (human)	NSCs (multilayered	Stable karyotype, proliferating NESTIN+,	not shown	BrdU, GFP	AFG	CdN, Put	Wakeman et al. (2014)
	and AAV-GDNF injections	adherent network NSCs)	GFAP+, SOX2, GFAP+,-βIII-					
			tubulin+, BLBP+, DCX+					
	parthenogenetic hESCs	NSCs (cGMP grade, current GMP)	Euploid karyotype, proliferating SOX2+,	10 × 10${}^{6}$ GFP+	GFP, RFP	AFG	CdN, Put, SN	Gonzalez et al.
			NESTIN+, MUSASHI+, CD133+, OCT4-,	cells (4 sites left)				(2015, 2016)
			SSEA4 free of mycoplasma and viral	10 × 10${}^{6}$ RFP+				
			contaminants					
	adipose stem cells (NHP)	DA neurons (combined	βIII-tubulin+, NEFM+, MAP-2+,	2 × 10${}^{6}$ cells/site	BrdU	rhesus	CdN, Put, SN	Zhou et al. (2013)
		with adenovirus expressing	NESTIN+, TH+, PITX3+,	(3 sites/hemisphere)				
		neurturin and TH)	LMX1B+, MSX1+, FOXA2+					

**Table 1:** Continued.

**(a)**: Grafted cell								
characteristics								
pre-graft.								
Grafted	Origin	Developmental	Characteristics	Grafted number	Labeling	Recipient	Grafted	References
cell type		differentiation stage				species	sites	
DA neurons	hESCs GFP+	DA neuron (day 25)	FOXA2+, βIII-	7.5 × 10${}^{6}$ cells/	GFP	rhesus	CdN, Put	Kriks et al. (2011)
			tubulin+, LMX1A+, NURR1+,	brain (3 sites/hemisphere)				
			PITX3+, TH+; novel DA					
			markers (TFF3, TTR, EBF1, EBF3)					
	hiPSCs	DA neurons (neurospheres:	βIII-tubulin+ (85 % TH+ at day 42)	4.8 × 10${}^{6}$ cells/brain	NO	cyno	Put	Kikuchi et al. (2011)
		day 28 and day 42 ±						
		DA differentiation)						
	hESCs	DA neurons (neurospheres:	Decreased LMX1A and EN1	4.8 × 10${}^{6}$ cells/brain	NO	cyno	Put	Doi et al. (2012)
		day 14–day 28 and day 35–42)	expression and increased NURR1+					
			and percentage of TH+ cells from					
			early to late neurospheres					
	hESCs	DA neurons (from NSCs)	βIII-tubulin+, TH+,	1 × 10${}^{6}$ cells/site	GFP	AFG	CdN, SN	Daadi et al. (2012)
			NURR1+, PITX3+, FOXA2+					
	ESCs (NHP)	DA neurons (day 41–44	βIII-tubulin+, TH+, BF1+	5 × 10${}^{6}$ cells/brain	NO	cyno	Put	Sanchez-Pernaute
		DA differentiation)						et al. (2005)
	ESCs (NHP)	DA neurons	24 % TH+, PAX2+, PTX3+,	3–6 × 10${}^{5}$ cells/side	BrdU	cyno	Put	Takagi et al. (2005)
		(day 21 neurospheres)	NURR1+, LMX1B+ release DA.					
	iPSCs (NHP)	DA neurons (day 42)	βIII-tubulin+ (37 %;	2.5 × 10${}^{6}$ cells/brain	GFP	rhesus	CdN, Put, SN	Emborg et al. (2013a)
			GABA+, 49 %; TH+ 16 %/.	(2.5 × 10${}^{5}$ in SN)				
			GIRK2+/FOXA2+/CALBINDIN)					
			S-100β+ (16 %),					
			NESTIN+ (50 %)					
	iPSCs (NHP)	DA neurons (day 30)	FOXA2+/TH+/βIII-	5 × 10${}^{6}/$site	NO	cyno	Put	Sundberg et al. (2013);
			tubulin (> 3 %),	(4 sites unilateral)				Hallett et al. (2015)
			TRA-1-81-, TRA-1-60-					
	iPSCs (NHP)	DA neurons (day 18–22)	βIII-tubulin+, TH+, FOXA2+,	not shown, Ren et al. (2013) =	GFP/Feridex	cyno	CdN, Put	Wang et al. (2015)
			NURR1+, GIRK2+	5 × 10${}^{6}$ cells/brain	nanoparticles			
	iPSCs (NHP)	DA neurons (day 28)	βIII-tubulin+ LMX1A, FOXA2	4.8 × 10${}^{6}$cells/brain	GFP	cyno, no MPTP	Put	Morizane et al. (2013)
				(8 × 10${}^{5}$/tract)				
Other cell types	human retina	human levodopa-	not shown	65–100 × 10${}^{3}$ cells/	NO	rhesus	CdN, Put	Peng et al. (2016)
		producing RPE cells		site (5 sites/brain) unilateral				
	NHP (females)	PKH26 endometrium-	not shown	4 × 10${}^{6}$ cells	PKH26	AFG	CdN	Wolff et al. (2015)
		derived cells		(2 sites) unilateral				
	bone marrow (NHP)	GDNF-MSCs	CD73+, CD105+, CD90+,	5 × 10${}^{6}$/brain	Feridex	cyno	CdN, Put, SN	Ren et al. (2013)
			CD106+, Stro-1+. Multipotent.		nanoparticles			
	carotid bodies (NHP)	striatal carotid body	not shown	not shown	NO	cyno	Put	Luquin et al. (2011)
		cell aggregates						
	3 mm${}^{3}$ of DLPFC	cultured cortical brain	shown in Brunet et al. (2009)	2–4 × 10${}^{5}$/site	PKH67	AFG	CdN, Put, SN	Bloch et al. (2014)
	of same monkey	cells (“ANCE”)						
**(b)**: Grafted cell								
characteristics								
post-graft.								
Grafted	Developmental	Survival	Localization	Characteristics	Overgrowth	Inflammation	References	
cell type	differentiation stage				proliferation	microglia		
						activation		
Fetal fVM	E74 (cell suspension)	300–3800 TH+ cells/brain	TH+	TH+	not shown	not shown	Annett et al. (1994, 1995)	
	late fVM	not shown	TH+	TH+	not shown	not shown	Redmond Jr. et al. (1986)	
	E40–E50 & late gestation	3500 TH+ cells/site (E44)	TH+	early fVM led to	not shown	not shown	Sladek Jr. (1993b);	
		550 TH+ cells/site (E49)		increased DA in the DA-depleted			Elsworth et al. (1996)	
				CdN (host derived); no DA increase				
				with late stages fVM				
	E44–47	not shown	not shown	TH+ 5–10 % increased	not shown	not shown	Elsworth et al. (1996);	
				dopamine transporter/control.			Collier et al. (1997)	
				Enhanced oxidative metabolism				
				(cytochrome oxidase activity)				
	E41–47	11 500 cells/brain	TH+	TH+, synaptophysin	not shown	not shown	Sortwell et al. (1998)	
	E40–42	8000 DA neurons/VM,	TH+	TH+, dopamine transporter	not shown	not shown	Sladek Jr. et al. (1998)	
		donor doubling number of						
		grafted cells does not						
		proportionally increase						
		cell survival						

**Table 1:** Continued.

**(b)**: Grafted cells							
characteristics							
post-graft.							
Grafted	Developmental	Survival	Localization	Characteristics	Overgrowth	Inflammation	References
cell type	differentiation stage				proliferation	microglia	
						activation	
	E43–47	1000 TH+ (late VM)	NO	TH+	not shown	not shown	Collier et al. (2002)
		10 000 TH+ (early VM)					
	E44–45	up to 100 000 TH+	TH+	TH+, GIRK2+, CALBINDIN+	not shown	minimal neuro-	Kordower et al. (2017)
						inflammatory response	
						(LN3/GFAP staining)	
	transfected with AAV5-	not shown	NO	TH labeling illustrations,	not shown	not shown	Redmond Jr. et al. (2013)
	hu-GDNF vector			striatal punches analyzed			
				for DA and GDNF concentration			
	E40	not shown	TH+	TH+ grafted cells acts via synaptic contacts	not shown	not shown	Leranth et al. (1998)
				on host neurons. The synaptic targets are			
				dendrites and somata of host neurons			
	E42–E45 (cell	12 000–60 000 surviving	TH+	TH+, GIRK2+, CALBINDIN+	not shown	stronger glial reaction	Redmond Jr. et al. (2008)
	suspension/solid pieces)	TH+ cells similar solid/					
		cell suspension and CdN/Put				to solid graft	
	various stages	< 500 TH+ cells/graft	TH+	TH+	not shown	no sign of rejection	Taylor et al. (1991)
	E38–44; E80–165	up to 18 000 TH+ cells	TH+	TH+ early fVM: increased DA	not shown	not shown	Taylor et al. (1996)
				levels and neuritic extensions			
	E45–47; E52–54	1500–6000 TH+ cells/graft	TH+	TH+/DAT+	not shown	not shown	Sladek Jr. et al. (2008)
		10 000 TH+ cells in the					
		SN when striatal graft					
		closest to the SN					
	not shown	not shown	TH+	TH+, NF70+, GFAP+ increased (18F)F-L-	not shown	weak IBA1/CD68/GFAP in	Aaron-Badin (2016)
				L-DOPA fixation with CTLA4-Ig		immunosuppressed animals	
NSCs	NSCs at early	up to 10 % surviving cell TH+:	BrdU labeling/	few neurons (3 % βIII tubulin+),	NO	no microglial activation	Redmond Jr. et al. (2007);
	passages (13 GW)	< 1 % of grafted cells	βGal staining	few TH+ cells GFAP+, GDNF+ migration		(18F-DPA714 binding)	Bjugstad et al. (2008)
	NSCs (multilayered adherent	16–41 mm${}^{3}$ graft TH+:	eGFP	< 20 % NESTIN+, 80 % NEUROFILAMENT+,	NO	no microglia	Wakeman et al. (2014)
	network NSCs)	< 1.1 % of grafted cells		no MAP2 and no DA differentiation;			
				few GFAP+; GDNF not sufficient to			
				induce DA differentiation			
	NSCs (cGMP grade)	10 % RFP+ cells engrafted TH+:	GFP or RFP	TH+, GIRK2+, VMAT2+, synaptophysin+;	NO	no microglia or host	Gonzalez et al.
		1.85 % of grafted cells		higher DA neuron innervation (TH fiber		microglia (IBA-1).	(2015, 2016)
				density) in striatum (low dose of cells)			
	DA neurons (combined	very low (few BrdU+ cells)	BrdU labeling	not shown	not shown	not shown	Zhou et al. (2013)
	with adenovirus expressing						
	neurturin and TH)						
DA neurons	DA neuron (day 25)	not shown	GFP human-	TH+, FOXA2+	not shown	persistent inflammation (IBA1+)	Kriks et al. (2011)
			specific SC-121				
	DA neurons (neurospheres: day 28 and	smaller graft size with day 42; TH+:	NO	NURR1+,VMAT2+, DAT+, GIRK2+,	low (< 1 % KI67+)	not shown	Kikuchi et al. (2011)
	day 42 ± DA differentiation)	1.25 % (day 28) 5.25 %		PITX3+; CALB+; Improved DA differentiation	day 28 > D42 grafts		
		(day 42) of grafted cells		(TH+) in day 42 grafts			
	DA neurons (neurospheres:	smaller grafts with day 14 grafts;	TH+ and DAT labeling	39 % NeuN+ (8 % TH+,	< 1 % KI67+ cells; decreased	not shown	Doi et al. (2012)
	day 14–28 and day 35–42)	13 000–18 000 TH+ cells/side		VMAT2+, AADC+, PITX3+); other neurons	tumorigenicity for day 42 grafts		
		(< 0.75 %TH+)		mostly GABAergic; increased DA synthesis (PET			
		higher DA yield with day 42		analysis of [18F] L-DOPA uptake) in the			
				grafts compared to host striatum			
				(linked to behavioral improvement)			
	DA neurons (from NSCs)	TH+: 10 % of the grafted cells	GFP human-	TH+; GIRK2+; CALB+; synaptophysin; serotonin-	not shown	not shown	Daadi et al. (2012)
			specific nuclei				
	DA neurons (day 41–44	TH+ cells: < 0.1 % of the grafted cells	NO	TH+, DAT+, AADC, few serotonergic,	NO	microglia (CD68+).	Sanchez-Pernaute
	DA differentiation)	(3300 TH+/brain); graft		few DCX+ neuroblasts.			et al. (2005)
		volume: 5.4–14 mm${}^{3}$		Increased F18 fluorodopa			
	DA neurons (day 21	8000 total cells/side (1.3–2.7 %)	BrdU labeling	TH+, DAT+, high percentage of GABA+	NO	not shown	Takagi et al. (2005)
	neurospheres)	4300 TH+cells/brain (0.4 %)		neurons, few serotonergic;			
		(underestimated due to BrdU labeling)		GIRK2+ CALB+			
	DA neurons (day 42)	less than 1.5 % total survival	GFP	MAP2+ (63 %), GFAP+(22 %),	NO (KI67-/OCT4-/NANOG-/	low inflammatory reaction	Emborg et al. (2013a)
				MBP+ (10 %); majority of MAP2+	SOX17-/BRA-)	and reactive microglia	
				are GABA+; few TH+			
	DA neurons (day 30)	13 000 TH+ cells/putamen:	TH+/FOXA2+	TH+/FOXA2+/βIII tubulin + cells	NO	low inflammatory reaction	Sundberg et al. (2013);
		functional improvement					Hallett et al. (2015)
	DA neurons (day 18–22)	20 000 TH+ cells whole brain	MRI	TH+; FOXA2+; NURR1+;	NO (KI67-)	NO (IBA1-)	Wang et al. (2015)
		(0.4 % if 5 millions were grafted)		GIRK2+; serotonin-			

**Table 1:** Continued.

**(b)**: Grafted cells							
characteristics							
post-graft.							
Grafted	Developmental	Survival	Localization	Characteristics	Overgrowth	Inflammation	References
cell type	differentiation stage				proliferation	microglia	
						activation	
	DA neurons (day 28)	autologous: 4400 TH+ cells/	GFP+	TH+ (higher number in autografts)	not shown	weaker immune rejection (lower nb of	Morizane et al. (2013)
		tract (0.5 % of grafted cells)				CD45+ CD8+, CD3+ cells) and activated	
		allogeneic: 2200 TH+/tract		(GIRK2?, CALB?), FOXA2+,		microglia (${}^{11}$C-PK11195 uptake;	
				NURR1+, DAT+, GFAP+		IBA1+) in autologous grafts	
Other cell types	human levodopa-	not shown	NO	Glucose metabolism	not shown	not shown	Peng et al. (2016)
	producing RPE cells			(18F-FDG PET imaging)			
	PKH26 endometrium-derived cells	very low	PKH26	TH+, (GIRK2?, CALB?)	not shown	not shown	Wolff et al. (2015)
	GDNF-MSCs	not shown	MRI	increased host DA levels, 5-HT in	not shown	no activation of	Ren et al. (2013)
				striatum/SPECT imaging (TRODAT1 uptake)		astrocytes and	
				no increase in host DA neuron survival		microglia	
	striatal carotid body	poor (80–100 TH+ cells/	NO	TH+	not shown	not shown	Luquin et al. (2011)
	cell aggregates	injection TH+site)					
	cultured cortical brain	SN (20–50 %), CdN (30–40 %),	PKH67	increased TH+ cells in grafted animals	not shown		Bloch et al. (2014)
	cells (“ANCE”)	Put (10–20 %)		(originating from the graft?)			

Abbreviations. C: cyclosporine; CPA: cyclosporine/prednisone/azathioprine; Put: putamen; CdN: caudate nucleus; NAcc: nucleus accumbens; SN: substantia nigra; cyno: cynomolgus; AFG: African green monkey.

Grafted cell survival can be improved through implantation of micrografts or cells over several sites within striatum or SN of NHPs (Table 1a; Collier et al., 2002; Redmond Jr. et al., 2007; Gonzalez et al., 2015, 2016; Hallett et al., 2015), confirming earlier rodent studies (Nikkhah et al., 1994). Another strategy to increase cell survival of grafted cells involves delivery of neurotrophic factors combined with cell transplantation (Elsworth et al., 2008; Redmond Jr. et al., 2013). In this context, glial cell-derived neurotrophic factor (GDNF) is one of the most widely tested neurotrophic factors, and it is well known to promote survival of DA neurons in vitro (Lin et al., 1993) and in vivo in different animal models of PD including NHPs, where it prevents degeneration of DA neurons (Kordower et al., 2000; Grondin et al., 2002; Elsworth et al., 2008). The neurotrophic actions of GDNF in PD have been extensively described elsewhere (Duarte et al., 2012; Sullivan and Toulouse, 2011; d'Anglemont de Tassigny et al., 2015). Neurexophilin 3 (NXPH3), a synapse-related peptide, may be also be used in combination with cell grafts for promoting cell survival. It was recently shown to support survival of mouse iPSC-derived DA neurons in vitro and in vivo, when combined with grafted cells (Nishimura et al., 2015).

#### Number of TH + cells required for functional recovery

Despite the low-cell surviving rate of the grafted cells, several studies showed behavioral improvement upon cell grafting in MPTP NHPs (Table 2). This suggests that a minimal restoration of normal DA innervation in the striatum is sufficient for functional recovery, as previously shown in the rodent model (Grealish et al., 2014). However, a certain threshold of TH+ cell dose is needed to improve PD motor symptoms and was estimated to be approximately 10 000 TH+ cells per brain, whether derived from ESCs (Doi et al., 2012) or from iPSCs (Hallett et al., 2015; Wang et al., 2015). A lower number of TH+ cells led to poor dopamine reinnervation and no functional recovery (Sanchez-Pernaute et al., 2005; Hallett et al., 2015). Considering that the monkey striatum is 5 to 7 times smaller than the human striatum (Yin et al., 2009), these results are in accordance with those obtained in humans, where 100 000 dopamine-producing cells isolated from fVM were necessary to reach optimal functional outcome (Lindvall, 2013).

**Table 2:** Characteristics and outcomes of the graft.

Species	Number	Grafted cells	Grafted sites	Immuno-	Survival time	Functional outcome	Ref.
	grafted			suppression			
Xenograft							
AFG	22	human NSCs	CdN/SN	no or C or CPA	4–8 months	motor score reduced by	Redmond Jr. et al. (2007);
						half at month 2 post-graft	Bjugstad et al. (2008)
rhesus	2	DA cells from hESCs	CdN/Put	C	1 month	no report of motor score evolution,	Kriks et al. (2011)
						no quantification	
cyno	1	neural progenitors	Put	FK506	6 months	no improvement in reaching task	Kikuchi et al. (2011)
		from hiPSCs				nor in neurological score; increasing	
						FDOPA uptake between months 3–6	
						post-graft; no significant change	
						in DAT binding vs. pre-graft	
cyno	9	DA cells from hESCs	Put	FK507	12 months	significant motor recovery	Doi et al. (2012)
						from month 3 post-graft only in	
						cases grafted with late (d42) DA cells;	
						FDOPA uptake significantly correlated	
						with degree of motor recovery	
AFG	4	DA cells from hESCs	CdN/SN	CPA	2 months	no report of motor score evolution	Daadi et al. (2012)
						and no quantification	
AFG	10	NSCs from hfVM and	CdN/Put (GDNF)	CPA	1.5 and 11 months	asymptomatic monkeys; some	Wakeman et al. (2014)
		AAV-GDNF injections	SN (hfVM-derived NSCs)			directional outgrowth of	
						grafted cells but no proof of	
						nigrostriatal reconstruction	
AFG	2	NSCs from human ESCs	CdN/Put/SN	C	14 weeks	asymptomatic monkeys	Gonzalez et al. (2015)
AFG	12	NSCs from human ESCs	CdN/Put	CPA	6 and 12 months	spontaneous motor recovery in non-grafted group;	Gonzalez et al. (2016)
						faster, more pronounced recovery and increased	
						DA, HVA in low-cell vs. high-cell group	
cyno	12	porcine fVM – solid blocks	Put	CPA	1–48 months	2–6 fold increased locomotor activity	Aaron-Badin et al. (2016)
						at month 6 post-graft vs. pre-graft and	
						significant increase in FDOPA uptake ratio	
						(grafted vs. non-grafted side)	
rhesus	6	human levodopa-producing	striatum	no	48 months	significant reduction in motor score (4/5 cases)	Peng et al. (2016)
		RPE cells				at month 6 post-graft; motor recovery maintained	
						up 48 months post-graft; no change in glucose	
						metabolism post-graft but PRP decreased	
						in grafted vs. non-grafted side	
AFG	22	human NSCs	CdN/SN	no or C or CPA	4–8 months	∼ 50 % reduction in motor	Redmond Jr. et al. (2007);
						score at month 2 post-graft; migration	Bjugstad et al. (2008)
						of NSC-derived TH+ cells to	
						contralateral non-grafted SN	
Allograft							
rhesus	2	DA cells from hESCs	CdN/Put	C	1 month	no report of motor score evolution	Kriks et al. (2011)
						and no quantification	

**Table 2:** Continued.

Species	Number grafted	Grafted cells	Grafted sites	Immuno-	Survival time	Functional outcome	Ref.
				suppression			
AFG	2	fVM – solid blocks	striata	no	69 days	parkinsonian score significantly reduced	Redmond Jr. et al. (1986)
						at 69 days post-graft and CSF-HVA concentration	
						at 40–80 % of baseline (non-grafted MPTP	
						cases ∼ 20 % of baseline)	
AFG	7	fVM – solid blocks	CdN	no	7–8 months	full recovery of motor score and healthy	Taylor et al. (1991)
						behavior at month 7–8 post-graft;	
						no recovery in sham	
AFG	3	fVM – solid blocks	CdN	no	14 weeks	no report of motor score evolution	Sladek Jr. et al. (1993b)
						post-graft; in some grafts a 4 to 14-fold	
						increase in DA concentration vs.	
						lesioned non-grafted striatum	
Xenograft							
marmoset	6	fVM – cell suspension	CdN/Put/NAcc	no	6 months	significant reduction in rotational	Annett et al. (1994)
						behavior at month 3 post-graft correlating	
						with number of surviving striatal TH+ cells;	
						no improvement in cognitive task	
marmoset	9	fVM – cell suspension	CdN or Put	no	10–12 months	better recovery of rotational behavior	Annett et al. (1995)
						for Put grafted cases vs. CdN grafted	
						cases at month 3 post-graft	
AFG	16	fVM – solid blocks	CdN or Put	no	9 months	severe parkinsonian cases CdN-grafted	Taylor et al. (1995)
						show more than 50 % reduction	
						on motor score at month 9	
AFG	20	fVM – solid blocks	CdN	no	18 months	only early stage fVM donor induce 50 %	Elsworth et al. (1996a)
						reduction in parkinsonian score coherent with	
						∼ 15–35 % increase in DA concentration	
						near grafts vs. non-grafted striatum	
AFG	3	fVM – solid blocks	CdN or Put	no	9 months	∼ 10 % increase in DAT	Elsworth et al. (1996b)
						binding and DA concentration near	
						grafts location accompanied by	
						slight behavioral improvement	
AFG	4	fVM – solid blocks	CdN or Put	no	8 months	no report of motor score evolution post-graft	Collier et al. (1997)
AFG	10	fVM – solid blocks	CdN or Put	no	6 months	no report of motor score evolution post-graft	Sortwell et al. (1998)
AFG	8	fVM – solid blocks	striatum	no	?	no report of motor score evolution post-graft	Sladek Jr. et al. (1998)
AFG	2	fVM – solid blocks	CdN	no	3 months	asymptomatic	Leranth et al. (1998)
						monkeys	
AFG	10	fVM – solid blocks	SN	no	6 months	significant but slight reduction in	Collier et al. (2002)
						parkinsonian score at month 5–6 post-	
						graft associated with modest increase	
						in DA concentration in ventrolateral Put	
cyno	1	ESC-derived DA cells	Put	no	7 months	no report of motor score evolution post-graft;	Sanchez-Pernaute et al. (2005)
						∼ 0.1 % survival on average	Sanchez-Pernaute et al. (2005)

**Table 2:** Continued.

Species	Number grafted	Grafted cells	Grafted sites	Immuno-	Survival time	Functional outcome	Ref.
				suppression			
cyno	6	ESC-derived DA cells	Put	C	14 weeks	50 % reduction in motor score	Takagi et al. (2005)
						after week 8 post-graft; FDOPA uptake	
						significantly increased at week 14 vs. sham	
AFG	6	fVM – solid blocks	Put/SN	no	11, 20 and 36 weeks	asymptomatic monkeys	Sladek Jr. et al. (2008)
AFG	20	fVM – solid blocks	CdN or Put	no	10 months	> 50 % reduction in	Redmond Jr. et al. (2008)
		or cell suspension				motor score after month 10 post-graft	
						for severe and moderate cases;	
						significant increase in healthy behavior score;	
						cell suspension grafts led to reduced GFAP+	
						cells in all cases and increased TH+ cells	
						at Put only; Put grafts better correlate	
						with motor improvement than CdN grafts	
rhesus	9	neuronal-primed ASCs	CdN/Put/SN	no	12 months	stable significant recovery in both	Zhou et al. (2013)
		and/or adenovirus expressing				motor score and rotational behavior	
		neurturin and TH				in combined group only at month 2 post-graft;	
						qualitative increase in striatal DAT levels at week 16	
						post-graft only in combined group;	
						coherent higher TH+ cells in SN of combined group	
AFG	13	fVM – solid blocks and/	CdN/Put	no	9 months	recovery in motor score for all grafted vs.	Redmond Jr. et al. (2013)
		or AAV5-hu-GDNF vector				sham at month 1 post-graft; combined graft	
						cases almost fully recovered at month 2 post-	
						graft in fVM only group and not complete	
						in vector only group	
AFG	16	PKH26 endometrium-	CdN	no	1 month	asymptomatic monkeys; ∼ 30 %	Wolff et al. (2015)
		derived cells				increase in HVA concentration in	
						grafted vs. non-grafted side	
Autograft							
rhesus	4	NSCs derived from	CdN/SN	no	5 months	no quantification reported;	Xu et al. (2010)
		MSCs and/or hTH				more reduction in motor score in cases	
						receiving hTH-NSCs and higher DAT levels	
cyno	7	CBCA	Put	no	12 months	no quantification reported for motor score,	Luquin et al. (2011)
						max recovery at month 6 post-graft;	
						FDOPA uptake significantly larger	
						vs. sham at month 12	
cyno	1	DA cells from iPSCs	Put	no	12 months	no quantification reported;	Sundberg et al. (2013)
						no change in motor score nor daytime	
						activity pre- vs. post-graft	
rhesus	3	DA cells from iPSCs	CdN/Put/SN	no	6 months	no quantification reported;	Emborg et al. (2013a)
						no obvious behavioral recovery nor	
						PET changes for VMAT2 binding	

**Table 2:** Continued.

Species	Number grafted	Grafted cells	Grafted sites	Immuno-	Survival time	Functional outcome	Ref.
				suppression			
cyno	8	DA cells from iPSCs	Put	no	3–4 months	no MPTP treatment; about twice	Morizane et al. (2013)
						the number of surviving cells	
						in auto- vs. allografts	
cyno	6	GDNF-expressing MSCs	CdN/Put/SN	no	8 weeks	MPTP given after grafts; neuroprotection	Ren et al. (2013)
						against MPTP for motor function of limb	
						contralateral to grafted side; DAT increase	
						in grafted side vs. non-grafted side	
cyno	3	DA cells from iPSCs	Put	no	12–24 months	1/3 case recovered daytime activity,	Hallet et al. (2015)
						as well as hypokinesia and increased DAT	
						level in grafted side after month 6 post-graft	
AFG	6	ANCE	CdN/Put/SN	no	6 months	only one case that received twice the amount	Bloch et al. (2014)
						of cells per site did not recover; 2 cases of	
						full and 2cases of partial motor recovery	
						in 200 days post-graft	
cyno	1	DA cells from iPSCs	CdN/Put/SN	no	6 months	motor recovery (significantly different	Wang et al. (2015)
						from non-grafted group) at week 6–8	
						and week 22–24 post-graft	

Abbreviations. C: cyclosporine; CPA: cyclosporine/prednisone/azathioprine; Put: putamen; CdN: caudate nucleus; NAcc: nucleus accumbens; SN: substantia nigra; cyno: cynomolgus; AFG: African green monkey.

The required number of surviving TH+ cells to reach functionality may also depends on the severity of DA loss. Indeed, imaging studies have shown that parkinsonian monkeys that recovered from motor symptoms following MPTP treatment show about 30 % more striatal DAT levels (Vezoli et al., 2014), about 20–35 % more striatal (fluorodopa) FDOPA uptake and 10–20 % more TH+ nigral cells than monkeys displaying stable parkinsonian motor symptoms (Blesa et al., 2012). In more severely affected monkeys, more graft-derived dopamine may be necessary to reverse parkinsonian behavior. On the contrary, in less affected monkeys, the host nigrostriatal DA system may still be capable of displaying a regenerative response after transplantation, and a lower cell number might then be necessary (Elsworth et al., 1996).

### 3.2 Progenies of the grafted cells in the host brain

The principal focus when analyzing the graft composition is on DA neurons. The most widespread marker of DA neurons is tyrosine hydroxylase, the enzyme that catalyzes the conversion of tyrosine to dihydroxyphenylalanine, which is the first step in the biosynthesis of dopamine. However, TH is also involved in the synthesis of other catecholamines such as epinephrine and norepinephrine. The identity of DA neurons is thus usually refined by studying the expression of DAT through immunostaining analyses (Sanchez-Pernaute et al., 2005; Takagi et al., 2005; Kriks et al., 2011; Hayashi et al., 2013; Morizane et al., 2013) or measurement of the binding potential of ${}^{11}$C-2β-carbomethoxy-3β-(4-fluorophenyl)tropane (${}^{11}$C-CFT) at the dopamine nerve terminals (Hayashi et al., 2013; Hallett et al., 2015). The graft content is occasionally further analyzed through staining for GIRK2 and CALBINDIN to highlight the presence of A9 and A10 DA neurons respectively (Kikuchi et al., 2011; Kriks et al., 2011; Sundberg et al., 2013; Wang et al., 2015).

As previously mentioned, PSC dopaminergic differentiation in vitro leads to variable amounts of TH+ DA neurons, as well as to other types of neurons, astrocytes and in certain cases neural progenitors. Accordingly, the content of the grafts from PSC-derived DA neurons is variable, with a usually high proportion of MAP2+ neurons, from which only a small proportion expresses TH (Takagi et al., 2005; Doi et al., 2012; Emborg et al., 2013a). A9 type DA neurons generally maintained their original A9 characteristics upon grafting (Hayashi et al., 2013; Wang et al., 2015). Non-negligible amounts of other types of neurons (GABA+ and SEROTONIN+), GFAP+ astrocytes and MBP+ oligodendrocytes are also found in the grafts (Emborg et al., 2013a).

NSCs, isolated either from fetuses or PSCs, generally poorly differentiated into TH+ neurons and principally gave rise to glial cells or undifferentiated neural progenitors when transplanted in MPTP-treated NHPs (Redmond Jr. et al., 2007; Bjugstad et al., 2008; Wakeman et al., 2014; Gonzalez et al., 2015, 2016). These non-neuronal cells may be involved in the reestablishment of adequate homeostasis in the lesioned brain, as previously suggested (Redmond Jr. et al., 2007). More recently, high-throughput RNA sequencing analyses enabled in-depth characterization of the grafts and showed that hNSC grafts induced the expression of genes and pathways that have been previously reported to be downregulated in PD (Gonzalez et al., 2016).

### 3.3 Axonal outgrowth and migration in the host brain

One of the critical issues of cell therapy in PD is the capacity of the transplanted cells to grow axons and reinnervate the DA-denervated host striatum over distances that are relevant for the size of the human brain. The adult brain has been suspected of no longer being capable of eliciting and directing axonal outgrowth from the SN to the striatum. Grafted cells have thus often been placed ectopically into the striatum, which is the site of lost dopaminergic input (Annett et al., 1994, 1995; Elsworth et al., 1996; Sanchez-Pernaute et al., 2005; Takagi et al., 2005; Kriks et al., 2011; Daadi et al., 2012; Doi et al., 2012; Morizane et al., 2013; Sundberg et al., 2013).

When transplanted in the lesioned rodent striatum, DA neurons from human fVM and PSCs show extensive reinnervation of striatal and extra-striatal target structures (Brundin et al., 1986; Sanchez-Pernaute et al., 2005; Thompson et al., 2005; Kirkeby et al., 2012; Grealish et al., 2014), and, when grafted in the SN, they project axons over long distances and reinnervate the relevant A9 and A10 host target structures (Grealish et al., 2014), as was observed for rodent DA neurons (Thompson et al., 2009).

In contrast, their capacity to reinnervate distant targets seems rather limited in the NHP brain, with TH+ fibers extending only a few millimeters into the host (Sladek Jr. et al., 1998; Collier et al., 2002; Takagi et al., 2005; Kriks et al., 2011; Emborg et al., 2013a), although this parameter was often not extensively documented (Elsworth et al., 1996; Sanchez-Pernaute et al., 2005; Takagi et al., 2005; Kikuchi et al., 2011; Daadi et al., 2012; Doi et al., 2012; Hayashi et al., 2013; Morizane et al., 2013; Sundberg et al., 2013; Hallett et al., 2015; Wang et al., 2015). fVM grafts performed either in the CdN or in the putamen induce increased TH innervation of the non-grafted ipsilateral nucleus (Redmond Jr. et al., 2008), suggesting that they could potentially extend long neurites. However, direct innervation of the remote striatum from SN grafts has not been observed in these species (Collier et al., 2002; Daadi et al., 2012; Emborg et al., 2013a; Ren et al., 2013; Bloch et al., 2014).

To promote reinnervation of the nigrostriatal circuitry, multiple intrastriatal and intranigral fVM grafts have been used as “bridge grafts” that attract the growth of neurites from grafted DA neurons in the rat (Mendez et al., 1996, 2000) and NHP models (Sladek Jr. et al., 1993a, 2008). Using this strategy, fVM grafts placed in the SN extended neurites over long distance preferentially to striatal co-grafts, suggesting that axon guidance cues are still present in the lesioned brain to guide the growing axons from the grafted DA neurons to their appropriate targets.

The nature of these guidance cues is still poorly known in the lesioned NHP brain. Several molecules such as the NETRIN, SLIT, EPHRIN and SEMAPHORIN families of secreted proteins, whose axon guidance activities have been extensively studied in the nervous system, have been shown to affect embryonic DA axons and may be involved in the regulation of axonal outgrowth of transplanted cells in the lesioned NHP brain. NETRIN-1 attracts whereas SLIT-2 repels rodent midbrain DA neurons in vitro and in vivo (Lin et al., 2005; Li et al., 2014), and both proteins affect human PSC DA neuron outgrowth in vitro (Cord et al., 2010). Signaling through the EPHRIN family receptor EphB1 and ligand EPHRIN-B2 is involved in the regulation of axonal growth of developing DA neurons in rodents (Yue et al., 1999; Sieber et al., 2004). EphA4 receptor and EPHRIN-B2 ligand are expressed in the adult NHP brain, including the CdN, putamen and SN (Xiao et al., 2006), and may play a role in directing axonal outgrowth of grafted cells. SEMAPHORINs are expressed in the rodent striatum and may be involved in the establishment of DA projections from the midbrain to the striatum during embryonic development (Hernandez-Montiel et al., 2008; Kolk et al., 2009; Torre et al., 2010). SHH signalling is involved in DA axon pathfinding and determination of the structural diversity of the DA projections during rodent development and may also promote axonal growth of grafted cells (Hammond et al., 2009). Whether these guidance cues persist in the adult or lesioned NHP brain is still unknown.

In addition to its action on DA neuron survival, GDNF also stimulates outgrowth of DA neurons after lesion or grafting in the rodent (Sinclair et al., 1996; Sautter et al., 1998; Wilby et al., 1999; Zhang et al., 2013) and NHP brain (Elsworth et al., 2008; Redmond Jr. et al., 2009; Wakeman et al., 2014). Combination of GDNF and NETRIN-1 was found to support directed long-distance growth of DA axons from rodent fVM grafts (Zhang et al., 2013). However, recent studies showed that GDNF delivery combined with fVM graft did not lead to increased functional improvement in the PD NHP model (Redmond Jr. et al., 2013), highlighting the need to clarify the benefit of GDNF delivery in this context.

Besides axonal outgrowth, migration of the transplanted cells is also involved in the reinnervation of the lesioned striatum over long distances.

In the rodent model, transplanted primate DA neurons extensively migrate, even reaching the contralateral hemisphere (Sanchez-Pernaute et al., 2005). In the NHP model, the sparse data available suggest that migration of DA neurons is limited, as judged by the lack of expression of the migrating neuroblast marker, doublecortin (Sanchez-Pernaute et al., 2005). In contrast to DA neurons, primate NSCs show widespread migration throughout the MPTP-lesioned NHP brain (Redmond Jr. et al., 2007; Bjugstad et al., 2008; Brunet et al., 2009; Gonzalez et al., 2016) as was previously shown in various rodent models (Fricker et al., 1999; Guzman et al., 2007). In particular, NSC progenies were observed migrating along the nigrostriatal pathway, from the caudate to the putamen (Bjugstad et al., 2008; Gonzalez et al., 2016). The phenomenon of migration of immature cells is exemplified by unilateral injections, where the grafted cells were found migrating to the opposite hemisphere (Bjugstad et al., 2008; Brunet et al., 2009). Interestingly, human NSCs spontaneously and preferentially migrate to the region of cellular loss over long distances in the lesioned PD NHP brain (Bjugstad et al., 2008), suggesting that migration is not a random event. The signals that direct NSC migratory pattern in the MPTP model are not known. Progenies of human NSCs express the chemokine receptor CXCR4, suggesting that chemokine-dependent mechanisms are involved in the regulation of their migration (Imitola et al., 2004; Kelly et al., 2004; Chang et al., 2013).

Immature cells develop very slowly in the host environment, and this may take several months before they generate terminally differentiated progenies, including fully functional mature DA neurons that extend projections in the nigrostriatal pathway. Studies of axonal outgrowth and cell migration are thus more appropriate in the NHP model, which allows long-term analyses as well as studies to assess the capacity of grafted cells to innervate the host brain over sufficiently long distances to provide good innervation of the remote putamen in primates.

### 3.4 Neurotrophic support and interaction with the pathological brain

From the earlier transplantation studies of adrenal medullar cells (Madrazo and Franco-Bourland, 1991) to the most recent studies of NSCs and DA neuron transplantation (Redmond Jr. et al., 2007), the functional effects of grafted cells were hypothesized to be obtained not exclusively by a cell-replacement mechanisms but also through diffuse release of neurotrophic stimuli and neuroprotective support on host circuitry (Li et al., 2005; Redmond Jr. et al., 2007; Bloch et al., 2014; Gonzalez et al., 2015, 2016; reviewed in Martino and Pluchino, 2006). NSCs can express and produce in situ a wide array of transmembrane and trophic molecules capable of promoting tissue repair. GDNF and BDNF are expressed in glial cells derived from the grafted NSCs, in both the rodent and NHP models, and may provide trophic support to the pathological host milieu (Redmond Jr. et al., 2007; Gonzalez et al., 2015).

Close physical associations have commonly been observed between grafted cells and host cells, indicating intercellular relationships. For example, DA neurons from transplanted fVM tissue or isolated from PSCs establish synaptic contacts with host striatal neurons by targeting dendrites and somata of spiny neurons (Leranth et al., 1998), as visualized by synaptophysin staining (Sortwell et al., 1998; Daadi et al., 2012; Wang et al., 2015). Differentiated progenies of hNSCs were also found in close contact with host TH+ neurons that showed increased cell body size as compared to DA neurons of the lesioned brain (Bjugstad et al., 2005; Redmond Jr. et al., 2007; Gonzalez et al., 2015).

### 3.5 Overgrowth and tumor formation

One major concern with regard to the use of PSCs-based therapy is the risk of overgrowth or development of tumors. In the NHP model, tumor formation or overgrowth was generally not detected following transplantation of PSC-derived DA neurons (Sanchez-Pernaute et al., 2005; Takagi et al., 2005; Emborg et al., 2013a; Sundberg et al., 2013; Hallett et al., 2015; Wang et al., 2015) or immature NSCs, as judged by the negative staining for Ki67, a marker of proliferative cells (Table 1b; Redmond Jr. et al., 2007; Bjugstad et al., 2008; Gonzalez et al., 2015, 2016). As expected, pluripotent nuclear (OCT4, NANOG) and membrane markers (TRA-1-81, TRA-1-60, SSEA4) were also not found in these grafts (Emborg et al., 2013a; Morizane et al., 2013; Gonzalez et al., 2015; Wang et al., 2015).

Proliferating cells have occasionally been detected after transplantation of hiPSC-derived DA neurospheres (Kikuchi et al., 2011; Doi et al., 2012). However, prolonged PSC DA differentiation in vitro enabled a drastic reduction of tumorigenicity (Doi et al., 2012) as previously shown in the rat (Brederlau et al., 2006).

However, depending on the protocol used to obtain DA neurons, the population of grafted cells may still contain immature proliferating cells that may, in some cases, be incapable of differentiating in the host tissue and eventually form tumors in vivo. The possibility of uncontrolled growth of the grafted cells in vivo should still be a matter of concern. This highlights the need for deep characterization of the grafted cells and long-term preclinical studies in the NHP model to validate the safety of each cell type.

## 4 Impact of the immune status of the host brain

The brain has long been positioned as immune privileged, but it is now well established that this privilege is not absolute and that immunological rejection processes can occur in the CNS (Lindvall, 1989; Cicchetti et al., 2003; Louveau et al., 2015). Transplanted tissue or cells might thus be recognized as foreign, leading to their rejection after transplantation in the brain. Fetal VM tissue expresses MHC antigens that can elicit an immune response in the case of host mismatch (Widner et al., 1989). Expression level of MHC molecules is low in human PSCs and NSCs (Drukker et al., 2002; Vagaska et al., 2016) but can rapidly be induced in inflammatory conditions or following differentiation in vitro (Drukker and Benvenisty, 2004; Vagaska et al., 2016).

Xenografts in the MPTP-treated NHPs have thus usually been performed under immunosuppression (Table 2). In the immunosuppressed environment, transplanted cells elicit only weak immune reaction, with minimal glial scarring and host IBA1+ microglia around the graft core (Redmond Jr. et al., 2007; Kikuchi et al., 2011; Kriks et al., 2011; Daadi et al., 2012; Doi et al., 2012; Gonzalez et al., 2015, 2016; Aron Badin et al., 2016).

In the case of allografts in NHPs, immunosuppression has exceptionally been used, and, when evaluated, immune response or inflammatory reaction was generally found to be weak (Table 1b).

However, direct comparison of allo- and autografts of iPSCs in healthy NHPs reported immune reaction in allografts, with the presence of host microglial cells that expressed MHC-II, IBA1+ cells, CD45+ leucocytes and CD8+ killer T cells in the grafts (Morizane et al., 2013). In contrast, in the case of autotransplantation, the immune reaction was only minimal with rare reactive microglial cells and a low number of MHC-II-expressing cells. TH+ cell survival rate was also higher in autografts than in allografts with immunosuppression and led to functional recovery (Sundberg et al., 2013; Hallett et al., 2015; Wang et al., 2015).

These recent studies suggest that immunosuppression can only be withdrawn in the autologous models (Emborg et al., 2013b; Morizane et al., 2013; Sundberg et al., 2013). Direct comparison of autograft and allografts in the lesioned NHP brain might ultimately confirm the efficacy and safety of autologous iPSC-derived or iDA cells.

## 5 Functional and clinical outcomes

In marmosets (*Callithrix jacchus*), parkinsonism can be modeled with MPTP, 6-OHDA or also through overexpression of α-synuclein (Yun et al., 2015). The MPTP model reproduces typical neurotransmitter loss; unilateral 6-OHDA lesions allow evaluation of the asymmetry of motor symptoms, such as rotational behavior; α-synuclein overexpression in the midbrain mimics the slow onset of motor symptoms and allows for the investigation of the so-called presymptomatic period before appearance of characteristic motor symptoms. Only the MPTP and 6-OHDA models have been used to assess cell-replacement therapy. In 1988, MPTP-treated marmosets (cumulative dose 11.3 mg kg${}^{-1}$ over 3 days, intraperitoneal) subsequently received unilateral and bilateral fVM grafts (Fine et al., 1988). Spontaneous locomotor activity as well as amphetamine-induced hyperactivity were increased in grafted monkeys compared to MPTP-treated controls and sham-grafted animals, suggesting graft-derived DA release into grafted striatum. In subsequent studies conducted by Dunnet and collaborators using 6-OHDA to induce hemiparkinsonism, symptoms were assessed with a battery of motor tasks including rotational behavior (spontaneous and amphetamine-induced). They showed that, 3 months after fVM grafts in the caudate nucleus (CdN), putamen (Put) and nucleus accumbens (NAcc), there was a significant reduction in rotational behavior (both spontaneous and amphetamine-induced) that correlated with the number of TH+ cells counted in the striatum. However, no improvement in the cognitive task was observed (Annett et al., 1994). In the follow-up experiment (Annett et al., 1995) it was shown that, after 3 to 6 months following fVM tissue grafts in Put only, animals had better motor recovery compared to those that received fVM grafts in CdN only. They finally showed that only fVM grafts derived from the youngest donor age were efficiently reducing amphetamine-induced rotations (Annett et al., 1997).

Experiments done in African green monkeys (*Cercopithecus aethiops sabaeus*) all rely on the model developed by Redmond and collaborators (MPTP intramuscular, IM, at 0.2 to 2.15 mg kg${}^{-1}$ typically injected every day over a 5-day period) and are all produced with his collaboration. This was the first team to induce efficient recovery of MPTP-induced parkinsonism with fVM grafts (Redmond Jr. et al., 1986), and they have been very prolific in that effort (see Table 1). Work from this lab has shown through very careful and detailed motor score evaluation that full motor recovery is seen 7–8 months after fVM grafts into CdN, but not in sham-operated animals (e.g., grafts into cortex; Taylor et al., 1991). A subsequent study (Elsworth et al., 1996) showed not full recovery but instead slight improvement on the clinical motor scale. They additionally described that, at the fVM graft sites, DA concentration was increased compared to the non-grafted side, sham-operated or control MPTP-treated cases (10–12 % of DA concentration of control animals compared to less than 1–2 % of controls in other cases). Collier and colleagues (2002) showed slight but significant behavioral improvement after transplant of fVM cells into rostral SN of both severely and moderately parkinsonian monkeys 5–6 months post-graft (Collier et al., 2002). This slight improvement was correlated with a slight but significant increase in DA concentration confined to the medial–lateral putamen. Later Redmond and collaborators demonstrated that fVM grafts done in the putamen result in a better correlation with motor improvement than those done in CdN and that the GFAP+ area was reduced following cell suspension compared to solid graft of fVM (Redmond Jr. et al., 2008). In 2013, they compared fVM grafts in combination with human GDNF vector or not (Redmond Jr. et al., 2013). This study demonstrated that behavioral recovery was accompanied by a 3-fold increase in DA concentration in animals receiving fVM+vector compared to fVM only, whereas animals receiving vector only did not recover. Bloch and collaborators (Bloch et al., 2014) performed bilateral autotransplantation of prefrontal cortex biopsies (ANCE) in CdN, Put and SN (2–4 × 10${}^{5}$ cells per site) of parkinsonian African green monkeys (5 MPTP dose of 0.45 mg kg${}^{-1}$, 82 days prior to grafts). All animals grafted with living cells recovered from motor symptoms (within 200 days post-graft), including one case that received disrupted cell grafts but with 1.5 % surviving cells, except for the animal that received twice the amount of cells which did not show any improvement. Postmortem analyses showed an average of 30 % cell survival, with better survival rate in SN (∼ 40 %) followed by CdN (∼ 30 %) and Put (∼ 20 %). Striatal TH+ cells at the grafted sites were higher in all animals grafted with living cells compared to sham-grafted animals (∼ 20 to 70 % of non-MPTP-treated controls). The most recent study used symptomatic MPTP monkeys with some additionally rendered dyskinetic with levodopa treatment and showed that bilateral putaminal grafts of fVM did not lead to graft-induced dyskinesia (Kordower et al., 2017). Redmond and collaborators also tested xenografts of human fVM tissue. In 2007, they transplanted undifferentiated human NSCs derived from fVM tissue (hfNSCs) unilaterally in SN and bilaterally in the CdN (1–9 × 10${}^{6}$ cells per site) of African green monkeys receiving a cumulative MPTP dose of either 2.25 mg kg${}^{-1}$ (severe parkinsonism) or 1.75 mg kg${}^{-1}$ (motor asymptomatic). Severely parkinsonian monkeys showed a significant recovery of motor symptoms compared to sham-operated ones within the first 2 months post-graft. Those animals presented cell migration such that the percentage of donor-derived TH+ cells was the same between grafted and non-grafted SN (∼ 7 % of total TH+ population), as well as the DA concentration. Additionally, the proportion of cells with α-synuclein aggregates decreased to less than 20 % after hfNSCs grafts compared to more than 80 % in non-grafted MPTP-treated animals (Redmond Jr. et al., 2007). They later confirmed that implanted hfNSCs migrated along the nigrostriatal pathway toward SN (Bjugstad et al., 2008). Gonzalez and collaborators (2016) tested hpNSC grafts in CdN and Put of severely parkinsonian monkeys (MPTP 2.15 mg kg${}^{-1}$ IM over 5 days) with different concentrations of cells (1 and 2 million cells per site, with low and high dose respectively) and triple immunosuppression (Gonzalez et al., 2016). They showed that animals transplanted with a low dose of cells displayed faster and greater recovery than those transplanted with a higher dose and had significantly reduced motor scores 12 months post-grafts. The low-dose group also presented higher levels of DA and metabolite concentrations than high-dose and MPTP-lesioned control groups.

In rhesus (*Macaca mulatta*) and cynomolgus (*Macaca fascicularis*) monkeys, MPTP is administered either acutely, leading to rapid nigrostriatal lesion and expression of motor symptoms, or chronically over weeks to months, inducing a progressive DA lesion and slowly evolving non-motor and motor symptoms. The main difference between these models is that acute MPTP models induce a topography of the DA lesion different than that produced by chronic MPTP intoxication, following which the ventral striatum is the most preserved structure (Perez-Otano et al., 1994) comparable to what is observed in PD patients (Gibb and Lees, 1991). Sanchez-Pernaute and collaborators grafted DA cells derived from ESCs (cyno-1 line) in the anterior and posterior part of the right Put of one parkinsonian cynomolgus monkey after repeated intravenous MPTP injections (0.3 mg kg${}^{-1}$, once a week for 16 weeks). This monkey did not show asymmetry of motor scores after unilateral graft (values not reported; Sanchez-Pernaute et al., 2005). During the same period Takagi and collaborators grafted bilaterally neural progenitors derived from ESCs in the Put of parkinsonian cynomolgus monkeys after repeated intravenous MPTP injections (0.4 mg kg${}^{-1}$ twice a week for a month on average). They showed that grafted monkeys (treated daily with cyclosporine) started to recover motor symptoms 4 weeks post-graft (significantly different from the sham-grafted group) and stabilized after week 10 (Takagi et al., 2005). Accordingly, fluorodopa uptake in the putamen was significantly increased in ESC-grafted animals compared to sham-operated ones. Kikuchi and collaborators (2011) bilaterally transplanted neural progenitors derived from human iPSCs (10${}^{5}$ cells per site) in the Put (6 sites per hemisphere) of one cynomolgus monkey 12 weeks after MPTP intoxication (0.4 mg kg${}^{-1}$ intravenous, IV, twice a week until persistent motor symptoms) and with immunosuppression. The graft size (estimated from anatomical MRI scans) increased tremendously from month 1 to month 12 post-graft. PET-scan binding values of a tumor tracer (FLT, 3${}^{\prime }$-deoxy-3${}^{\prime }$-[${}^{18}$F]-fluorothymidine) also constantly increased, and, while still below control values, some Ki67+ cells were found in the graft showing proliferation of immature cells (< 1 %). Neither improvement in neurological score nor in reaching task performance could be seen. Doi and collaborators (2012) bilaterally transplanted hESC-derived neural progenitors at different stages (D14 and D28 floating spheres, D35 and D42 attached spheres) into the Put of parkinsonian cynomolgus monkeys (0.4 mg kg${}^{-1}$ MPTP IV until persistent motor symptoms). The D14 grafts contained up to 30 % of Ki67+ cells, and the uptake ratio of PET tracer (FLT) used to detect brain tumor was also substantially higher than control, indicating tumorigenicity, whereas D42 grafts contained less than 1 % of Ki67+ cells and an FLT uptake ratio comparable to control. However, even if FDOPA uptake was correlated with the neurological score change after graft, there was no significant recovery compared to sham-injected controls (Doi et al., 2012). Zhou and collaborators grafted rhesus neuronal-primed adipose stem cells (ASCs) combined or not with gene transfection of neurotrophic factor and TH in CdN, Put and SN of hemiparkinsonian rhesus monkeys after single intracarotid MPTP injection (0.6 mg kg${}^{-1}$). Animals showing stable apomorphine-induced rotational behavior for more than 12 months were used for transplantation. Only the combined group showed stable signs of recovery (significant reduction in motor score, i.e., close to non-MPTP-treated controls, and, in apomorphine-induced rotational behavior) as well as increased levels of striatal DAT 4 months post-graft (no quantification). A progressive recovery was observed in the group transplanted with neuronal-primed ASCs only, and, coherently, the TH+ immunoreactive cells counted in the SN were 15 % of the intact side. TH+ cells rose to 30 % of the intact side in the combined group, thus providing evidence that about 30 % of TH+ cells in the SN is enough to induce stable recovery (Zhou et al., 2013). Xu and collaborators (2010) performed autografts of NSCs derived from BMSCs and transfected or not with human gene coding for TH in CdN and SN (1.5–3 × 10${}^{6}$ cells per site) of hemiparkinsonian monkeys 6 weeks following 1-month intoxication with intracarotid perfusion of MPTP (1.2 mg kg${}^{-1}$; Xu et al., 2010). Hemiparkinsonian monkeys transplanted with NSCs showed behavioral improvement with better amelioration in clinical score for groups transplanted with NSCs modified to express human TH (no report of scores or degree of recovery). Five months following grafts, striatal FDOPA uptake and DAT levels were tested and showed respectively an increase of grafted side compared to control lesioned monkeys and higher values of DAT levels for grafted group with NSCs expressing hTH (qualitative, no values or statistical tests reported). EGFP+ and TH+ double-labeled cells were found in SN of all grafted groups, with higher proportion in grafted group with NSCs expressing hTH (no values reported for cell counts). Some years later, MSCs modified or not to express GDNF were grafted unilaterally in CdN, Put and SN (∼ 5.2 × 10${}^{6}$ cells per site) of cynomolgus monkeys (Ren et al., 2013). MPTP intoxication was performed 2 weeks after grafts (0.1 mg kg${}^{-1}$ IV) until animals displayed stable scores (moderate to severe, 10–14 on the PPRS) and bilateral upper limb function was assessed through a food retrieval task. DAT levels were also measured before and 6 weeks after MPTP intoxication. The GDNF+-MSCs provided neuroprotection against MPTP for motor function (better performance on the limb contralateral to grafted side), and, accordingly, DAT levels as well as DA and DA metabolites levels were significantly increased in the grafted side compared to the non-grafted side. There were no neuroprotective effects seen in the MSCs-only graft group. Luquin and collaborators (Luquin et al., 2011) performed unilateral and bilateral carotid body cell aggregates (CBCA) in the rostral and caudal Put of cynomolgus monkeys 3 months after stable expression of severe to moderate parkinsonism following weekly intoxication with MPTP (0.05–6 mg kg${}^{-1}$). FDOPA uptake was controlled 1 week before surgery and at 6 and 12 months post-surgery. The maximum motor recovery was observed at 6 months post-grafts and stabilized (no report about degree of recovery). After grafts, FDOPA uptake showed a tendency to decrease in sham-operated animals, whereas the grafted group showed a tendency to increase. The FDOPA uptake at 12 months was significantly higher in grafted compared to sham-operated animals. The authors reported a significant increase of TH+GDNF+ cells in the SN of the grafted group, suggesting trophic factors release from putaminal CBCA grafts.

Remaining studies all used iPSCs mainly in cynomolgus monkeys. Emborg and collaborators (2013) implanted iPSCs in the anterior and posterior parts of CdN; in the anterior, mid- and posterior parts of Put; and in SN (2.5–5 × 10${}^{5}$ cells per site) of hemiparkinsonian rhesus monkeys 12 to 18 months following MPTP intoxication by intracarotid infusion (Emborg et al., 2013b). There was no obvious behavioral recovery and no PET change (data not shown) following transplantation. Hallet and collaborators (Hallett et al., 2015) transplanted unilaterally iPSC-derived DA neurons 2–4 years after induction of stable parkinsonism in Put (4 sites, 1–4 × 10${}^{6}$ cells per site) of cynomolgus monkeys treated with intravenous injections of MPTP (0.15–0.3 mg kg${}^{-1}$, 1–2 times a week for more than 10 weeks). Recovery in motor score (and notably hypokinesia) and daytime activity was observed in only one of the three grafted cases for which DAT levels were estimated with a PET scan and showed an increase in the grafted side compared to the non-grafted side. Sundberg and collaborators transplanted iPSC-derived DA neurons (Sundberg et al., 2013) in one cynomolgus monkey treated with intravenous injections of MPTP (0.3 mg kg${}^{-1}$, once a week for 5 weeks until expression of mild stable parkinsonism). Neither motor score nor daytime activity recovery was observed following transplantation. Wang and collaborators injected iPSCs derived from one SFV-infected monkey (simian foamy virus, SFV, has been shown to interfere with iPSCs production) rendered hemiparkinsonian through unilateral intracarotid infusion of 3 mg of MPTP (Wang et al., 2015). Behavioral recovery was observed at two time points following transplantation – i.e., clinical score significantly differed compared to non-grafted cases at weeks 6–8 and again after week 22 post-graft. TH+ cells were detected in grafted Put and CdN, but not in grafted SN. Finally, Peng and collaborators (2016) transplanted unilaterally human retinal pigment epithelial cells into the CdN–Put (5 tracks 2 mm apart along the rostrocaudal extent of the caudal CdN–Put, 6 to 10 × 10${}^{4}$ cells per site) of mild to moderately severe parkinsonian rhesus monkeys (MPTP intravenous, over several months) and followed them behaviorally and evaluated changes in the parkinsonism-related pattern (PRP) of glucose metabolic activity measured with PET imaging (Peng et al., 2016). Clinical improvement was seen after 6 months in all grafted animals and stable over a 2 to 4-year period of follow-up, whereas no improvement in the clinical rating scale could be seen for sham-grafted animals. The PRP network activity was increased after MPTP and significantly reduced following graft while still remaining above the values of age-matched healthy controls.

NHP models of PD are all reproducing motor symptoms characteristic of the disease, degeneration of nigrostriatal DA neurons and linked perturbation of functional markers, e.g., reduction in FDOPA uptake and/or DAT binding. However, only some models can reproduce the early onset of cognitive and circadian disturbances also present in PD and display a slowly progressing nigrostriatal lesion, e.g., repeated low-dose MPTP. Therefore, both the model and the palette of reproduced parkinsonian symptoms are of importance for appropriate translation to the clinic. Multiparametric longitudinal monitoring of functional and behavioral consequences of cell-replacement therapy is thus crucial for efficient translation to the clinic. More than a third of the reviewed literature (see Table 2) did not report any in vivo functional, e.g., with PET-scan, nor behavioral outcome of the grafts (6 studies used asymptomatic MPTP monkeys and one non-MPTP-treated monkeys), focusing exclusively on postmortem evaluation of host–graft integration (Sladek Jr. et al., 1993b, 1998, 2008; Collier et al., 1997; Leranth et al., 1998; Sortwell et al., 1998; Sanchez-Pernaute et al., 2005; Bjugstad et al., 2008; Redmond Jr. et al., 2009; Kriks et al., 2011; Daadi et al., 2012; Morizane et al., 2013; Wakeman et al., 2014; Gonzalez et al., 2015; Wolff et al., 2015). In these conditions it is impossible to infer the degree of integration required for any behavioral or functional recovery. However, even if those studies are necessary for testing different types of transplant before investigation in a larger number of animals, reporting clinical motor score has been very well documented for many parkinsonian models and should be an imperative standard, considering that those investigations require sacrifice of NHPs. From the remaining studies, about half of them reported in vivo functional outcome following graft in addition to behavioral outcome assessing clinical motor symptoms; however, some studies just reported qualitatively those functional changes (Xu et al., 2010; Emborg et al., 2013a; Sundberg et al., 2013; Zhou et al., 2013). Surprisingly, and even so non-motor symptoms are recognized as having a great impact on quality of life in PD patients (Simuni and Sethi, 2008), no graft study using the NHP MPTP model to date has reported effects on non-motor symptoms. We found one study in the 6-OHDA marmoset model reporting effects of fVM grafts on a version of the detour task used to assess integrity of DA innervation to the frontal cortex (Annett et al., 1994). Assessing non-motor symptoms has just lately being explored in a rodent model of PD (Lelos et al., 2016) and will hopefully pave the way for systematic follow-up of non-motor symptoms in cell-repair research for PD.

## 6 Perspectives

Grafting in NHPs will enable the development of techniques for more detailed in vivo and postmortem follow-up of the fate of the cells that can be translated to humans. Because NHP and human cells share the same characteristics and cell signalling regulations, NHPs are potentially the most appropriate for in vivo screening.

However, the burdens from working with NHPs are significant in terms of time and cost investments needed to pursue preclinical investigations and/or provide significant contribution to scientific knowledge about the underlying mechanisms of PD. Limitations are often required by ethic committees which follow a country's directives; for example, recently the European Commission asked the Scientific Committee on Health, Environmental and Emerging Risks (SCHEER) to update the opinion on the use of nonhuman primates in researchhttps://ec.europa.eu/health/scientific_committees/consultations/public_consultations/scheer_consultation_03_en SCHEER provides recommendations, after public consultation, on how to advance training, improvement of techniques and protocols, sharing of knowledge, removal of barriers and research needs for NHP use.. Progress was made, but it was not enough to justify a reduction in the use of NHPs in neurodegenerative disease research. Rather, both advances in recent promising techniques and the need for more preclinical design studies in NHPs call for a controlled increase in the use of NHPs in neurodegenerative research. In return, rigorous individual follow-up with pertinent functional imaging evaluations during the different intoxication and transplantations phases (pre/post) should be standard practice for preclinical observations.

We have seen that site of transplantation is critical for behavioral recovery. Studies originally placed transplants into the striatum, the target of DA neurons for practical reasons, and showed that placement into Put lead to better motor recovery compared to caudate transplants (Annett et al., 1995; Redmond Jr. et al., 2008). Multiple small transplants (e.g., Peng et al., 2016) are also an efficient solution to implant more cells, but not in one single location, which has been showed to be detrimental (Bloch et al., 2014; Gonzalez et al., 2016). Combination of multiple target areas might promote reconstruction of the nigrostriatal pathway but still needs further refinement in NHPs. No clear evidence of nigrostriatal reconstruction has been shown so far, neither with combined grafts in striatum and SN (Redmond Jr. et al., 2007; Bjugstad et al., 2008) nor in SN grafts with trophic factors in the striatum (Wakeman et al., 2014). However, interhemispheric cell migration is possible (Redmond Jr. et al., 2007; Bjugstad et al., 2008), and transplanted cells favor the natural nigrostriatal connectivity patterns for selective neuritic outgrowth (Wakeman et al., 2014). Indeed, basal ganglia circuitry and especially nigrostriatal and striato-cortical connectivity patterns are well described in NHPs (Williams and Goldman-Rakic, 1998; Haber, 2003; Raghanti et al., 2008). According to these schemes, it is more than likely that grafts placed at given striatal sub-compartments would influence the specific system they support – e.g., graft placed in the caudal CdN might be more efficient in affecting cognitive troubles, whereas putaminal grafts might correlate better with motor recovery. With increased understanding of growth and guidance molecules affecting DA neurons, it may be feasible to place transplants in the damaged SN and direct the growth of axons into target regions for reconstruction of midbrain DA circuitry. Our established and ongoing understandings of the molecular cues which support directed growth of DA neurons form an important basis for the refinement and optimization of grafting procedures. Adding supporting factors for survival and axonal outgrowth of grafted cells (GDNF, NXHP3) and combining different cell types (multipotent NSCs, GDNF-producing cells) will be the next step in the refinement of the technique.

The latest developments in the field of neural tissue engineering could not be implemented here, but shall certainly be taken into account in future transplantation strategies in PD NHPs. Various biocompatible and biodegradable biomaterials are currently being developed and may enhance survival and integration of the grafted cells (Lins et al., 2016; reviewed in Sensharma et al., 2017).

While the DA system is not the only neurotransmitter system altered, DA remains central in PD. DA plays an essential role in (i) multiple cerebral functions of the frontal lobe, e.g., in performance monitoring or in motivational aspects of behavior; (ii) motor function via the extrapyramidal system; and (iii) circadian regulation of behavior through interactions with lateral habenula and locus coeruleus. This multidimensional aspect of DA should trigger a multiparametric follow-up of the NHP model of PD. Longitudinal and simultaneous multiparametric follow-ups (cognitive and behavioral tasks, motor score, rest–activity cycles, in vivo imaging) should be carried out as much as possible given the NHP PD model chosen in future experiments. Indeed, non-motor symptoms have only been reproduced through repeated low-dose MPTP regimen (Schneider and Kovelowski, 1990; Taylor et al., 1990; Almirall et al., 1999, 2001; Barcia et al., 2003; Decamp and Schneider, 2004; Vezoli et al., 2011; Fifel et al., 2014). Overall, very few studies follow up transplanted monkeys for more than a year (Elsworth et al., 1996; Hallett et al., 2015; Aron Badin et al., 2016; Peng et al., 2016). However, a longer outcome period should be considered (2–4 years post-implantation) in order to assess potential long-term side effects, graft rejection or changes in functional in vivo measures, especially in cases that present good motor recovery. All these criteria will enable full validation of the safety and efficiency of cell grafting before clinical translation.

Finally, it should be noticed that recently emerging NHP models of PD, e.g., overexpression of α-synuclein (Marmion and Kordower, 2017), might be able to reproduce the slowly progressive DA lesion observed in PD without the need to repeat MPTP injections; however, they require further characterization before being used to evaluate cell-replacement therapy.
